# Antimicrobial and Biofilm Inhibiting Potential of Two Romanian Linden Honeys

**DOI:** 10.3390/foods14213594

**Published:** 2025-10-22

**Authors:** Alexandru Nan, Mihai Mituletu, Gabi Dumitrescu, Ion Valeriu Caraba, Ioan Pet, Adrian Sinitean, Mariana Adina Matica, Petculescu Chiochina Liliana, Elena Pet, Roxana Popescu, Marioara Nicoleta Caraba

**Affiliations:** 1 Doctoral School “Engineering of Vegetable and Animal Resources”, University of Life Sciences “King Mihai I” from Timisoara, Calea Aradului 119, 300645 Timisoara, Romania; alexandru.nan@usvt.ro; 2 Faculty of Bioengineering of Animal Resources, University of Life Sciences “King Mihai I” from Timisoara, Calea Aradului 119, 300645 Timisoara, Romania; valeriucaraba@usvt.ro (I.V.C.); ioanpet@usvt.ro (I.P.); lilianapetculescuciochina@usvt.ro (P.C.L.); 3 ANAPATMOL Research Center, “Victor Babes” University of Medicine and Pharmacy of Timisoara, E. Murgu, 2, 300041 Timisoara, Romania; popescu.roxana@umft.ro (R.P.); nicoleta.caraba@umft.ro (M.N.C.); 4 Faculty of Medicine, “Victor Babes” University of Medicine and Pharmacy Timisoara, E. Murgu, 2, 300041 Timisoara, Romania; 5 Department Biology, Faculty of Chemistry-Biology-Geography, West University of Timisoara, Pestalozzi, 16, 300315 Timisoara, Romania; adrian.sinitean@e-uvt.ro (A.S.); mariana.matica@e-uvt.ro (M.A.M.); 6 Advanced Environmental Research Laboratories (AERL), 4 Oituz, 300086 Timisoara, Romania; 7 Faculty of Management and Rural Tourism, University of Life Sciences “King Mihai I” from Timisoara, Calea Aradului 119, 300645 Timisoara, Romania; elenapet@usvt.ro

**Keywords:** honey, polyphenols, flavonoids, microelements, antibacterial

## Abstract

Honey is a traditional remedy, with its biologically active compounds being responsible for its properties. The aim of this study was to characterize linden honey from a physico-chemical point of view as well as its antimicrobial and antibiofilm potential. Two samples of linden honey with different origins were subjected to physico-chemical analyses, including the determination of water content, impurities, dry matter, acidity, pH, reducing sugar content, total phenol content, flavonoids, antioxidant potency by DPPH, and mineral content. The microbiological analysis involved determining the inhibition rates of microbial growth and the antibiotic capacity of linden honey against ten standardized bacterial strains and five bacterial strains isolated from patients. The analyzed linden honey can be characterized based on physico-chemical parameters as having a slightly increased water content, moderate acidity, rich in antioxidants, and a balanced pH. The average concentrations of macroelements and microelements in the honey samples showed that potassium was the dominant mineral element, followed by calcium and magnesium. The heavy metal content was consistent with European and international standards. The chemical content of linden honey influenced its antimicrobial and antibiofilm potential. In Gram-positive bacteria, inhibition rates were between 70.83 and 91.28% (sample A) and 71.14–90.16% (sample B) when applying concentration c1. For Gram-negative bacteria, values ranged between 63.91 and 78.30% (sample A) and 46.56–90.92% (sample B) at concentration c1. In bacterial strains isolated from patients, the inhibition rate values were between 75.42 and 85.69% (sample A) and 78.31–86.22% (sample B) when applying concentration c1. The antimicrobial and antibiofilm potential was highlighted in all bacterial strains studied, with differences occurring depending on the concentration of honey tested and the type of bacterial strain studied.

## 1. Introduction

Honey is a natural sweetener that is widely available around the world and is used for its nutritional and therapeutic properties. The world’s oldest medical texts mention the medicinal value of honey. Since ancient times, it has been known to have the property of healing wounds, this being a result of its antibacterial properties [1,2].

Bee honey contains about 200 substances, mainly represented by water, sugars, and other substances such as proteins (enzymes), organic acids, vitamins (especially vitamin B6, thiamin, niacin, riboflavin, and pantothenic acid), minerals (potassium, calcium, magnesium, copper, iron, manganese, phosphorus, sodium, and zinc), pigments, phenolic compounds, a wide variety of volatile compounds, and derived solid particles, which contribute to the quality and health benefits of honey [3,4,5].

Honey is a supersaturated sugar solution in which monosaccharides account for about 75%, especially fructose and glucose, with their proportions in honey being between 32 and 44% (fructose) and 23–38% (glucose). The composition in sugars depends on the botanical origin of the honey (the types of flowers used by the bees), the geographical origin, the climate, the way of processing, and storage [6,7].

The mineral content of honey is generally low—between 0.02 and 0.03 g/100 g in the case of floral honey and up to 1–1.03 g/100 g in honeydew honey [8]. The mineral content is influenced by a multitude of factors, such as the nature of the raw material from which the honey comes, the content of mineral substances in the soil on which the honey flora grows, the degree of impurity of the raw materials, the method of processing and preserving the honey, etc. [9]. One of the main elements found in honey is potassium, which represents about 80% of the total minerals. In addition, other minerals were also identified, such as calcium, magnesium, phosphorus, chlorine, sulfur, iron, manganese, and silicon, and the spectral analysis revealed salts of aluminum, boron, copper, sodium, zinc, nickel, lead, osmium, tin, etc. In general, dark honey contains more minerals than light honey, with the concentration of minerals being higher in honeydew honey [10]. In addition, both ash content and electrical conductivity are related to the mineral concentration in honey [8].

Phenolic compounds contain approximately 10,000 constituents present in honey in highly variable amounts ranging from 5 to 1300 mg/kg [11,12]. Depending on their chemical structure, they are divided into non-flavonoid compounds (e.g., phenolic acids) and flavonoids (flavones, flavonols, flavanones, flavanols, anthocyanidin, isoflavones, and chalcones) [13]. The phenolic compounds and flavonoid profile of honey allow the evaluation of its quality, facilitating the differentiation of varieties, the establishment of botanical origin, as well as the detection of adulteration and bioactive compounds with sanogenic properties [14].

The physico-chemical properties and biological activity of honey can be maintained for a longer period of time only if certain storage conditions are met. According to the norms of the Russian Interstate Standard “Natural honey. Technical conditions”, whose requirements are similar to those laid down in the Codex Alimentarius and Council Directive 2001/110/EU, honey must be stored at a temperature below 20 °C, in spaces inaccessible to sunlight, packed in hermetically sealed containers, for 12 months from the date of examination and 24 months from the date of packaging [15]. It was found that exposing honey to low temperatures (from 0 to −20 °C) for a longer period of time slows down chemical processes and stabilizes some physico-chemical parameters such as humidity, electrical conductivity, acidity, and content in phenolic compounds [16]. In addition, storing honey at low temperatures slows down the formation and accumulation of hydroxymethylfurfural (HMF), a toxic product resulting from the dehydration of monosaccharides, which has important cytotoxic, neurotoxic, genotoxic, and mutagenic effects [17,18,19].

Honey has unique nutritional and medicinal properties. The numerous therapeutic effects of honey are known, including antioxidants [20], anti-inflammatory [21], antimicrobial [22,23,24,25], antiparasitic [26], wound-healing capacity [2], anticancer [27], treatment of eye diseases [2], treatment of gastrointestinal tract diseases [2], treatment of neurological diseases and disorders [28,29], antidiabetic action [30], and treating heart diseases [31]. All these therapeutic properties are due to the physical properties and chemical composition of honey, which fluctuate depending on the plants from which the bees collect the raw material, the climatic and geographical conditions, the harvesting, storage, and storage conditions [2].

So, the chemical composition of honey, its taste, and its color depend mainly on its botanical origin, the species of bees that produced the honey, the climate, and the geographical region, but they can also be influenced by weather conditions and its processing, packaging, and storage [3]. The therapeutic potential of honey is attributed to its antioxidant and antimicrobial capacity [32], with polyphenols being partially responsible for the antioxidant activity of honey [33]. Flavonoids and polyphenols present in honey samples act as antioxidants against free radicals, preventing cell aging. There is usually a correlation between the total content of polyphenols and flavonoids and the antioxidant capacity of honey [20], but this is not always the case because the antioxidant capacity of each sample is the combined result of other non-phenolic compounds [34].

Although the mechanisms of antibacterial action of honey are not fully elucidated, honey is known for its antimicrobial effects, having a wide spectrum of action against microorganisms. Numerous studies show the broad spectrum of antimicrobial activity of honey against Gram-positive and Gram-negative microorganisms, but the results obtained are contradictory [22,24,35,36,37]. It is assumed that the antimicrobial property of honey is influenced by factors such as high carbohydrate content, osmolarity, H_2_O_2_ content, low pH, polyphenol and flavonoid content, 1,2-dicarbonyl methylglyoxal (MGO), and defensin 1 [38,39,40].

Many of the components of honey are responsible for its antibacterial effect, including the high concentration of sugars and high osmotic pressure [41]; H_2_O_2_ and the high concentration of the compound 1,2-dicarbonyl methylglyoxal (MGO) [38]; the concentration of phytochemicals, especially phenolic compounds [40]; glucose oxidase enzyme concentration and defensin-1 concentration [39]; the presence of non-protein components in honey that does not contain H_2_O_2_ [42]; and the combination of unidentified anionic and cationic substances other than those shown [41].

Since the results presented in the studies are contradictory regarding the contribution of phytochemicals to the antimicrobial activity of honey, further studies are needed with different varieties of honey from different geographical regions and testing strains of microorganisms both in vitro and in vivo.

The purpose of the work is the physico-chemical characterization and evaluation of the antibacterial and antibiofilm potential of an assortment of linden honey from the western part of Romania. The work represents a preliminary in vitro study to highlight the antimicrobial and antibiofilm activity of linden honey against Gram-negative and Gram-positive bacterial strains, standardized and isolated from patients.

## 2. Materials and Methods

### 2.1. Honey Samples

**Figure 1:**
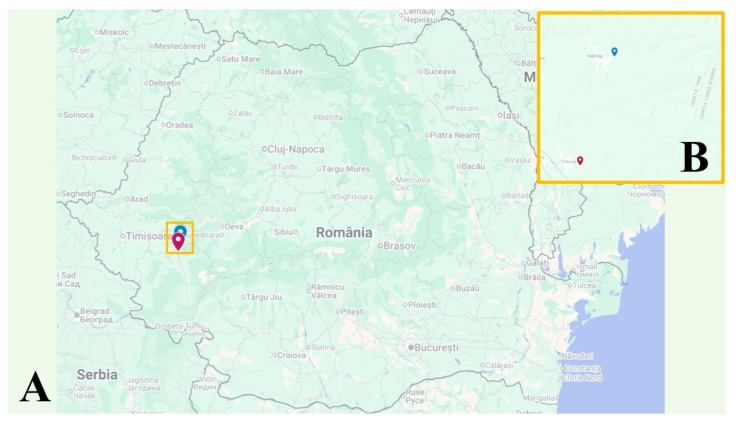
Location of the apiary ((**A**)—general, (**B**)—detail) (after Google My Maps, 19 October 2025) Coordinates: Sample A (red) 2021: 45°34′11.95″ N, 22° 9′35.23″ E; Sample B (blue) 2021: 45°39′7.02″ N, 22°12′8.32″ E).

The analyzed honey type was represented by linden honey (*Tilia* sp.) sourced from an apiary located in a hilly area: sample A—near the village of Tincova (45°34′11.95″ N, 22°9′35.23″ E, western part of Romania) and sample B—near the town of Nadrag (45°34′11.95″ N, 22° 9′35.23″ E, western part of Romania) (Figure 1). The tested honey samples (sample A and sample B) represent the single sample obtained after randomly collecting and mixing honey from 10 different samples taken from the entire quantity of linden honey obtained per season. The honey samples came from an organic apiary, which holds certification for honey quality, with more than 5 years of experience in the beekeeping field of the beekeeper. The honey comes from the beekeeping production of 2021. The honey samples were transported and stored in glass jars at a temperature of ≈20 °C in a dry, dark place. The results presented are those obtained following the analyses carried out between September and December 2021. The chemical analyzes were carried out at the Interdisciplinary Research Platform (PCI) belonging to the University of Life Science “King Mihai I” from Timisoara, Romania, and the microbiological analyzes were carried out at the Advanced Environmental Research Laboratories (AERL) belonging to the West University of Timisoara.

### 2.2. Palynologic Assessment of the Honey Samples

The honey assortment was determined based on palynological analysis. A total of 20 mL of distilled water was added to a 10 g sample of honey; the contents were homogenized on a Biosan MR-1 oscillating shaker (Biosan, Riga, Latvia) and subsequently placed in a water bath at 40 °C to ensure the dissolution of sugars. The prepared sample was then placed in the MPW-351RH centrifuge and centrifuged at 3500 rpm for 15 min (MPW Med. Instruments, Warsaw, Poland). The obtained sediment was transferred onto dry glass slides, embedded in glycerin gel, and covered with coverslips. The obtained slides were subjected to microscopic analysis using a Nikon Eclipse TE2000-U inverted microscope (Nikon Instruments, Amstelveen, The Netherlands). For each sample, 500 pollen grains were counted and identified based on morphological characters (size, shape, exine characteristics) [43,44].

### 2.3. Determination of Impurities

Impurities (such as beeswax, pollen, tiny bee parts, etc.) can enter the honey during the spinning and packaging process. The tested honey samples (10 g/sample) were homogenized for 30 min with the help of a stirrer, after being previously dissolved in 50 mL of water. After homogenization, the samples were weighed, passed through filter paper, and placed in an oven to dry the paper for 10 min at 103 °C. After drying, the residues left on the paper were weighed, and the impurities were calculated according to the formula:

I = (m1/m2) × 100 (%),

where I represents the amount of impurities (%); m1 represents the initial mass of the sample taken for analysis (g); and m2 represents the mass of the residues remaining on the filter paper after drying (g) [45]. The results are expressed in 100 mg/100 g honey.

### 2.4. Determination of Humidity

Moisture was determined by drying 5 g/sample at 103 °C for 24 h using the conventional oven drying method (Binder GmbH, Tuttlingen, Germany). After drying, the samples were weighed, and the percentage of the moisture was determined using the formula:

Moisture = [(G1 − G2)/G1 − G3)] × 100 (%)

Dry matter = 100 − Moisture (%)

where G1 represents the weight of the Petri dish and the sample before drying (g); G2 represents the weight of the Petri dish and the sample after drying (g); and G3 represents the weight of the Petri dish (g) [46].

### 2.5. Determination of Acidity

The acidity of the honey samples was analyzed and determined by the titration method. Ten grams of each honey sample was dissolved in 50 mL of water to which 2 drops of alcoholic phenolphthalein solution were added. For complete dissolution, the samples were stirred for 30 min using the Holt IDL plate shaker, Freising, Germany, then passed through filter paper and titrated with 0.1 N sodium hydroxide solution (until pink color persisted for 30 s). The acidity was calculated by applying the formula:

Acidity = [(V × 0.1)/10] × 100 (mL NaOH 0.1 n/100 g honey)

where V represents the volume of sodium hydroxide solution used in the titration (mL) and 0.1 is the normality of the sodium hydroxide solution used in the titration [46].

### 2.6. Determination of pH

The measurement of the pH of the honey samples was carried out at a temperature of 23–24 °C, using an inoLab pH 730 pH meter (Xylem Analytics, Weilheim, Germany) at a pH working range of −2.000 ± 19.999, with a precision of ± 0.05. For sample preparation, 3 g of honey/sample was used, which was dissolved for 30 min in 30 mL of water, using the Holt Stirrer LM4-1002 plate stirrer (IDL, Freising, Germany) [46].

### 2.7. Determination of Reducing Sugar

The determination and evaluation of the reducing sugar level were carried out according to the method described by Lazar et al. [45]. An amount of 3 g of honey/sample each was distributed in graduated flasks, over which water was added up to 200 mL and mixed. To obtain the working solution, 20 mL/sample was transferred to new containers, and the difference up to 100 mL was filled with water. Separately, 20 mL of copper sulfate solution, 20 mL of alkaline Seignette salt solution, and 20 mL of water were mixed, and the mixture was heated to near boiling point, when 20 mL of the working solution was added. The mixture was boiled for 5 min, and after it was cooled in a water bath, 25 mL of sodium chloride solution was added, gently shaking the vessel, until the contents became clear with a bluish-green appearance. At the end, 2 g of sodium bicarbonate was added, and after a few minutes, an intense blue sodium bicarbonate residue was observed at the bottom of the vessel. Iodine solution was gradually added to the obtained solution, stirring constantly. Initially, the color of the solution was milky white, then it became clear, and finally green. To identify excess iodine, 0.5 mL of starch solution was added to the solution, the color varying from green to dark blue. The solution was again titrated with sodium thiosulfate until it turned light blue.

For the calculation of the reducing sugar expressed in inverted sugar, the formula was applied as follows:

Inverted sugar = [(m × 10 × 5)/(m1 × 1000)] × 100 (%)

where m represents the amount of inverted sugar (mg); m1 represents the amount of analyzed honey (g); 10 is the ratio between the volume of the solution in the volumetric container of 200 mL and the volume of the solution taken for dilution; and 5 is the ratio between the volume of the solution in the volumetric container of 100 mL and the volume of the diluted solution taken for analysis [46]. The results of the reducing sugar content are given in percentage (%).

### 2.8. Determination of Total Phenolic Content (TPC)

One gram of honey sample was weighed, over which 10 mL of 70% alcohol was added, homogenized for 30 min using the Holt Plate Stirrer (IDL, Freising, Germany), and then passed through filter paper. For 0.5 mL of the filtered sample, 1.25 mL of Folin–Ciocalteu reagent (Sigma-Aldrich Chemie GmbH, Munich, Germany) was added, diluted 1:10 with distilled water. The samples thus obtained were incubated at room temperature for 5 min. An amount of 1 mL of Na_2_CO_3_ (60 g/L aqueous solution) was added to each sample, and then the samples were placed for 30 min in the incubator (Memmert GmbH, Schwabach, Germany) at a temperature of 50 °C. Finally, the absorbance was read at 750 nm with a UV-VIS spectrometer (Analytical Jena Specord 205, Jena, Germany) using ethanol as a blank sample. The calibration curve was obtained using gallic acid (concentration range: 2.5–250 µg/mL), and the results were expressed in mg GAE per g dry matter (d.m.). Determinations were performed in triplicate [45].

### 2.9. Determination of Flavonoid Content (FC)

To determine the flavonoids, 10 containers with lids were prepared, in which 1 g of honey sample and 10 mL of 60% alcohol were inserted. Samples were then homogenized using a Holt plate Stirrer for 30 min, after which they were filtered using filter paper. Then, 1.5 mL of the prepared extract, 4.5 mL H_2_O, and 1 mL NaNO_2_ were added to 10 clear glass containers and incubated for 6 min. After incubation, 1 mL of Al(NO_3_)_3_ 10% was added, and the samples were incubated again for 6 min. Then, 10 mL NaOH 4% were added and supplemented with alcohol 70% up to 25 mL. After 15 min, the absorbance was read at 510 nm using a UV–VIS spectrometer (Analytical Jena Specord 205, Jena, Germany). Quercetin solution was used as control. Results were expressed in mg QE/100 g, and all determinations were performed in triplicate [24].

### 2.10. Determination of Antioxidant Capacity (DPPH)

The antioxidant capacity of the honey samples was determined with the DPPH method. Honey samples were weighed: 1 g of honey/sample, diluted with 10 mL of 60% alcohol, and then filtered. Obtained extracts were then incubated for 30 min. Samples were read on a UV–VIS spectrometer (Analytical Jena Specord 205, Jena, Germany) at the absorbance of 518 nm. Ethanol 60% was used for negative control. For positive control, 1 mL of DPPH solution (3 mM) and 2.5 mL extract were introduced in a test tube, incubated for 30 min, and then measured at 518 nm. The blank was 1 mL of ethanol and 2.5 mL of extract incubated for 30 min and measured at 518 nm.

The antioxidant activity of each sample was calculated using the following formula:

AA = 100 − [(A sample − A blank) × 100/A control]

where AA represents the antioxidant activity; A sample represents the absorbance of the sample; A control represents the absorbance of the DPPH sample; and A blank represents the absorbance of the alcohol sample [24].

### 2.11. Determination of Mineral Substance Content (Ash)

To determine the content of the mineral substance (ash), honey samples were distributed, 3 g/crucible each; honey samples were gradually heated and maintained at 550 ± 25 °C in the calcination furnace (Nabertherm, Lilienthal, Germany) until a white or light gray ash was obtained. The ash crucibles were subsequently weighed, and the results were obtained by applying the following formula:

Ash = (m − m1)/(m2 − m1) (%)

where m represents the mass of the crucible with the ash obtained after calcination (g); m1 represents the mass of the empty melting pot (g); and m2 represents the mass of the melting pot with honey (g).

An amount of 10 mL of hydrochloric acid was added to each sample; then, the contents were transferred to glass tubes, and the micro- and macroelement content was determined. After filtration, the samples were distributed into 50 mL volumetric flasks and made up to the mark with water. A multi-element standard solution, Centipur Merk, was used for calibration. The method used to quantify the main macro- and microelements was atomic absorption spectroscopy (AAS) [46,47]. The macro- and micromineral content of the analyzed honey was in mg/kg.

### 2.12. Antimicrobial Activity

Antimicrobial activity testing was carried out using 5 concentrations of each variety of honey (c1–c5), standardized microbial strains (ATCC), and bacterial strains isolated from patients. The raw honey obtained from the apiary represented the corresponding honey c1 (100%). Aqueous extracts from each honey sample were prepared by mixing 1 mL of honey with 1 mL of sterile distilled water; then, the other 4 tested concentrations of 80%, 60%, 40%, and 20% were made. The microbial reference strains (ATCC) used in this study were obtained from the culture collection of the Microbiology Laboratory of the Advanced Environmental Research Laboratories (AERL) belonging to the West University of Timisoara. The bacterial strains isolated from the patients were obtained from the Microbiology Department of the “Pius Brânzeu” Emergency Clinical Hospital in Timisoara.

#### 2.12.1. Bacterial Strains

Honey samples were tested on the following reference strains: Gram-positive strain, *Staphylococcus aureus* (ATCC 25923); *Staphylococcus aureus* MRSA (ATCC 43300); *Enterococcus faecalis* (ATCC 29212); *Streptococcus pneumoniae* (ATCC 49619); and *Streptococcus pyogenes* (ATCC 19615), and Gram-negative strains, *Shigella flexneri* (ATCC 12022); *Escherichia coli* (ATCC 25922); *Salmonella typhimurium* (ATCC 14028); *Hemophilus influenzae* type B (ATCC 10211); and *Pseudomonas aeruginosa* (ATCC 27853). The bacterial strains isolated from the patients were *Staphylococcus aureus*, *Staphylococcus aureus* MRSA, *Streptococcus pneumoniae*, *Streptococcus pneumoniae* beta-lactam resistant (*S. pneumoniae* β-lactam-R), and *Escherichia coli*.

#### 2.12.2. Bacterial Cultures

These standardized bacterial strains were previously cultured for 24 h at 37 °C in Trypticase Soy Broth Medium (TSB). Bacterial strains isolated from patients were transferred to new Trypticase Soy Broth Medium (TSB) and incubated for 24 h at 37 °C. After 24 h of incubation the optical density (OD) of each bacterial culture was measured at 620 nm using the BioTek Synergy/H1 microplate reader (Agilent Technologies, Santa Clara, CA, USA). The appropriate dilution for each bacterial strain was then made to reach an optical density (OD) of 0.5 McFarland standard (1.5 × 10^8^ CFU × mL) using TSB, and the inoculum was assessed with a McFarland densitometer (Grand-Bio, London, UK). The bacterial inoculum thus obtained will be the one that will be used to test the antibacterial potential [48].

#### 2.12.3. Bacterial Cell Viability Testing

Evidence of bacterial cell viability was performed by assessing the respiratory activity of microbial cells through the TTC test. Tetrazolium salts are reduced by the dehydrogenase enzyme and converted into formazan, which is a measure of the total respiratory activity of the cells. An amount of 100 µL of microbial cultures in Trypticase Soy Broth Medium (TSB) (with turbidity equivalent to 0.5 McFarland standard) was transferred into 96-well plates. A 50 µL solution of honey at concentrations c1–c5 was loaded into wells; then, plates were incubated at 37 °C for 24 h with 120 rpm. After incubation, 50 µL of 2,3,5-triphenyltetrazolium chloride 0.5% (TTC) solution was added into wells, and the plates were incubated at 37 °C for another 2 h with 120 rpm. Absorbance reading was performed at a wavelength of 460 nm using the microplate reader (BioTek Synergy/H1 microplate reader, Agilent, USA). The rate of inhibition (expressed as percentage) was determined using the following formula:

Rate of inhibition (%) = [(OD control − OD samples)/OD control] × 100

where OD sample represents the microbial population treated with concentrations c1–c5 of honey, and OD control represents the microbial population [49,50].

All experiments were conducted in triplicate.

#### 2.12.4. Inhibition Rate of Biofilm Formation

Evidence of the effect of honey on the ability to inhibit biofilm formation in the bacteria of interest was achieved using a modified version of the method proposed by Knezevic and Petrovic [51]. In this protocol, 100 μL of the bacterial inoculum was distributed in each well of the 96-well plate; then, 50 μL of honey was applied in the established concentration. The plates were incubated for 24 h at 37 °C, and after incubation, the liquid was removed and the plate was washed twice with sterile 0.9% NaCl solution, which was dried at 37 °C. Subsequently, 200 μL of 0.4% crystal violet was added to each well and allowed to act for 1 h at 37 °C. Excess crystal violet solution was removed by washing with tap water; then, 200 μL of 30% acetic acid was added to each well of the plate and left to act for 30 min. Absorbance reading was performed at a wavelength of 570 nm using the microplate reader (BioTek Synergy/H1 microplate reader, Agilent, USA). The determination of the biofilm formation inhibition rate was made with the following formula:

Biofilm inhibition rate (%) = (OD sample/OD control) × 100

where OD sample represents the microbial population treated with concentrations c1-c3 of honey, and OD control represents the microbial population. All experiments were conducted in triplicate.

### 2.13. Statistical Analyses

All determinations were made in triplicate, and the results are reported as mean values ± standard deviation (SD). The dendrogram (performed with PAST 4.03) is a tree diagram that displays the groups—in this case, the concentrations of minerals in honey—according to the level of similarity [52]. Principal component analysis (PCA) is a technique that reduces the number of dimensions in data while minimizing information loss. PCA prioritizes the principal components by importance, with PC_1_ being the component that explains the most variation in the data, followed by PC_2_, and so on. By considering only the first few principal components, such as the first two, a significant percentage of the variance in the data can be explained. This allows high-dimensional data to be represented on a two-dimensional plot. In the interpretation of loading vectors (“loadings”) in PCA, their magnitude shows how much each original variable contributes to the corresponding main component, as follows: (a) very high contribution, high loading (as close as possible to ± 1; > ±0.7); (b) high contribution, high loading (from ±0.5 to ±0.7; (c) moderate loading (from ±0.3 to ±0.5); and (d) small loading (<±0.3) [53]. Antimicrobial activity rates, chemical data, figures, and statistical analyses were performed using Microsoft Excel 365 and Past4.03. Differences between groups were assessed using T tests; differences were considered to be significant at *p* < 0.05.

## 3. Results

### 3.1. Palynological Analysis

The determination of the linden honey assortment for the honey samples subjected to analysis was made following microscopy analyses and based on the percentage of pollen grains belonging to the species *Tilia* sp. Relevant microscopic images from the palynological analysis, which formed the basis for the creation of Table 1, are presented in Supplementary Material Figure S1. At the level of the analyzed honey samples, the percentage of pollen grains was 47.85% for sample A and 51.75% for sample B.

In Table 1, the percentages of pollen grains identified at the level of the analyzed samples are shown. 

**Table 1:** Palynological analysis of the analyzed linden honey samples.

	Species	Asteraceae	Tiliaceae	Lamiaceae	Fabaceae	Rosaceae	Boraginaceae	Others
Honey Sample								
Sample A		12.15%	47.85%	10.35%	8.75%	7.50%	6.60%	6.80%
Sample B		11.62%	51.75%	3.25%	9.25%	8.25%	7.5%	8.38%

Palynological analysis revealed that the pollen belonging to the genus *Tilia* (linden) predominated, confirming that the honey samples analyzed came from geographical areas where linden trees are predominantly found.

### 3.2. Physico-Chemical Characterization

Honey is a highly complex food, with a diverse composition due to the components present in plant nectar or in the secretions coming either from plants or from sap-sucking insects. The physico-chemical properties of linden honey, determined based on the previously described analysis methods, are presented in the form of average and standard deviation in Table 2.

The statistical analysis highlights differences between all physico-chemical properties of linden honey from sample A and sample B, respectively, with these differences being statistically significant (*p* < 0.05) at WC (*p* = < 0.0001), IC (*p* = 0.0015), A (*p* < 0.0001), RS (*p* = 0.0268), and TPC (*p* < 0.0001).

**Figure 2:**
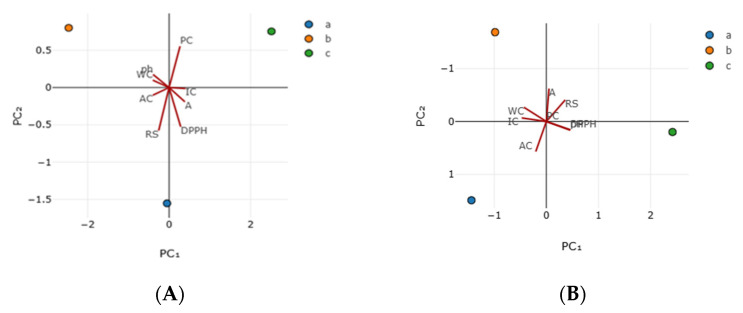
PCA biplot of the physico-chemical properties of the analyzed linden honey. ((**A**).—honey sample A (near the village of Tincova), (**B**).—honey sample B—(near the town of Nadrag)).

Principal component analysis (PCA) of linden honey from the point of view of physico-chemical properties is based on the correlation matrix, with standardized values. Since these properties (variables) have different units of measurement and can be represented graphically, an individual PCA analysis was carried out for each type of honey, A and B, respectively (Figure 2A,B).

For the physico-chemical properties of linden honey sample A, PC_1_ has an eigenvalue of 6.19, explains 77.4% of the total value (Table 3), and is the most important component of the recorded values. For the physico-chemical properties of linden honey sample B, PC_1_ has an eigenvalue of 4.45, explains 63.54% of the total value (Table 3), and is the most important component.

The highest loadings of PC_1_ were positive (they increase with the increase of PC_1_) if IC (loading 0.402) and A (loading 0.388) and, respectively, negative (they decrease as the component increases) for WC (loading −0.398), AC (loading −0.398), and pH (loading −0.391), all with a moderate contribution (from ±0.3 to ±0.5) (Table 3). For PC_1_, the IC and AC vectors are opposed to the WC, AC, and pH vectors, as confirmed by Figure 2A.

PC_2_ has an eigenvalue of 1.8 and explains 22.6% of the variance in the data. PC_2_ has a higher positive loading (increases with increasing PC_2_) for the TPC (loading 0.556) and a negative loading (decreases with increasing PC_2_) for RS (loading −0.576) and DPPH (loading −0.525), all with a high contribution (from ±0.5 to ±0.7) (Table 3). The TPC vector is in opposition to the RS and DPPH vectors, as also confirmed by Figure 2A.

PC_1_ and PC_2_ are thus sufficient to explain 100% of the variability in the data, and so we can reduce the size of the basis set without losing meaningful information. Therefore, secondary components PC_3_-PC_8_ are not considered to be significant.

For the physico-chemical properties of linden honey sample B, PC_1_ has an eigenvalue of 4.45, explains 63.54% of the total value (Table 3), and is the most important component. PC_1_ has higher positive loadings (increasing with the components) for pH (loading 0.459), DPPH (loading 0.457), and RS (loading 0.361) and, respectively, negative for IC (loading −0.471) and WC (loading −0.429), all with a moderate contribution to this component (from ±0.3 to ±0.5) (Table 3). The pH, DPPH, and RS vectors are in opposition to the IC and WC vectors of the PC1 for the honey sample B, as also confirmed by Figure 2B.

PC_2_ has an eigenvalue of 2.55 and explains 36.46% of the variation in the data. AC contributes with a moderate positive loading (0.566)—increases with the increasing components—to PC_2_ of honey sample B, while A contributes with a large negative loading (−0.622) and RC with a moderate negative loading (–0.407) to PC_2_. The AC vector is in opposition to the A and RS vectors (see Figure 2B).

Since it has a higher charge (vector 1) of WC, AC and pH (negative values) vs. IC and A (positive values) probably separate the data based on chemical characteristics. DPPH antioxidant activity contributes moderately to secondary components. PC_1_ and PC_2_ are thus sufficient to explain 100% of the variability in the data, and so we can reduce the size of the basis set without losing meaningful information. Therefore, secondary components PC_3_-PC_8_ are not considered to be significant. So, the linden honey sample analyzed can be characterized as having a slightly increased water content, with a moderate acidity, rich in oxidants and with a balanced pH.

**Figure 3:**
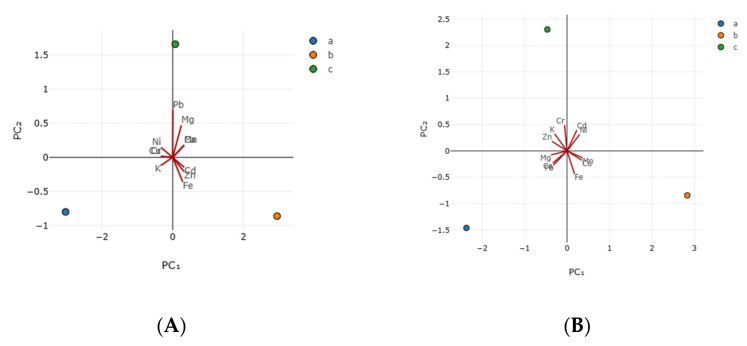
PCA biplot of mineral composition of honey. ((**A**).—honey sample A (near the village of Tincova), (**B**).—honey sample B—(near the town of Nadrag)).

The PCA of linden honey from the point of view of mineral composition is based on the covariance matrix, with standardized values, since these properties (variables) have the same units of measure (mg/kg) and can be represented graphically; also, an individual PCA analysis was carried out for each type of honey, A and B, respectively (Figure 3).

The physicochemical properties of linden honey sample A can be explained by two principal components: PC_1_ with moderately positive loadings for IC and A and moderately negative loadings for WC, AC, and pH. The associated vectors (IC and AC opposite to WC, AC, and pH) show the two trends of PC_1_. PC_2_ has a large positive loading for TPC and a large negative loading for RS and DPPH, which is confirmed by the opposition between the TPC vector and the RS and DPPH vectors.

The physico-chemical properties of honey sample B can also be explained by two principal components. PC_1_ has moderately positive loadings for pH, DPPH, and RS and moderately negative loadings for IC and WC, with the associated vectors pH, DPPH, and RS opposing IC and WC. In PC_2_, AC contributes moderately positively, while A contributes with a large negative loading and RC with a moderate negative loading. The vectors corresponding to AC are opposite to the vectors for A and RS.

For the mineral composition of linden honey sample A, the most important component PC_1_ has an eigenvalue of 8.94 and explains 81.22% of the data variation. PC1 represents higher positive loadings (loading increases with PC_1_) of Cd (loading 0.327; heavy metal, nephrotoxic and bone toxic; possible environmental contamination), Mn (loading 0.323; essential trace element), Ca (loading 0.323; which in large quantities are essential for vital body functions), and Zn (loading 0.319; essential micronutrients) and, correspondingly, higher negative loadings (loading decreases as the PC_1_ increases) of Cu (loading −0.335; essential micronutrients), Cr (loading −0.335; Cr(III) is an essential micronutrient essential in glucose metabolism, but Cr (IV) is heavy metal), K (loading −0.329; alkali metal), and Ni (loading −0.327; essential micronutrient); all these loads are larger, but they have a moderate contribution to PC_1_ (from ±0.3 to ±0.5). The second principal component PC_2_ has an eigenvalue of 2.07 and explains 18.78% of the variance in the data. PC_2_ (vector 2) has a higher loading, positive values (loading increases with PC_2_) in Pb (loading 0.696; heavy metal, neurotoxic; possible environmental contamination) and Mg (loading 0.472; alkali earth metal) and, correspondingly, higher negative loadings (loading decreases as the PC_2_ increases) in Fe (loading −0.361; transition metal beneficial in anemia and other redox process); Pb has a high contribution (from ±0.5 to ±0.7) at PC_2_, while Mg and Fe have moderate contributions (from ±0.3 to ±0.5) (Table 4).

For the mineral composition of linden honey sample B, PC_1_ has an eigenvalue of 6.93, explaining 62.96% of the total value (Table 4). The largest loads, but with a moderate contribution to PC_1_ (from ±0.3 to ±0.5), are either positive (increasing with the rise of PC_1_) for Mn (loading 0.366) and Cu (loading 0.354) and, respectively, negative (decreasing as the PC_1_ increases) for Mg (loading −0.376), Zn (loading −0.354), and Pb (loading −0.319). PC_2_ for the honey sample B has an eigenvalue of 4.08, explaining 37.04%. The largest loads, however, with a moderate contribution to PC_2_ (from ±0.3 to ±0.5), were positive for Cr (loading 0.489), Cd (loading 0.394), K (loading 0.321), and Ni (loading 0.310), and negative for Fe (loading −0.438) (Table 4).

The mineral composition of linden honey sample A can be summarized by two main components. PC_1_ has moderately positive loadings for Cd, Mn, Ca, and Zn and moderately negative loadings for Cu, Cr, K, and Ni. PC_2_ has a large positive loading on Pb and moderately positive loadings on Mg and moderately negative loading on Fe. The opposing associated vectors for PC_1_ are Cd, Mn, Ca, and Zn versus Cu, Cr, K, and Ni, while the corresponding opposing vectors for PC_2_ are Pb and Mg versus Fe. The mineral composition of the linden honey sample B can also be summarized by two main components. In PC_1_, Mn and Cu contribute moderately positively, and Mg, Zn, and Pb contribute moderately negatively. In PC_2_, Cr, Cd, K, and Ni have a moderate positive contribution, while Fe contributes negatively. The corresponding opposing vectors that characterize PC_1_ for honey B are Mn and Cu versus Mg, Zn, and Pb, and for PC_2_, the associated opposing vectors are Cr, Cd, K, and Ni versus Fe.

### 3.3. Antioxidant Activity

Polyphenols have an important antioxidant action, being involved in the elimination of free radicals [54]. At the level of linden honey subjected to analysis, the content of total polyphenols was 862.32 mg/kg (sample A) and 871.33 mg/kg (sample B), and the DPPH value was 80.39% (sample A) and 77.51% (sample B).

### 3.4. Antimicrobial Activity

**Figure 4:**
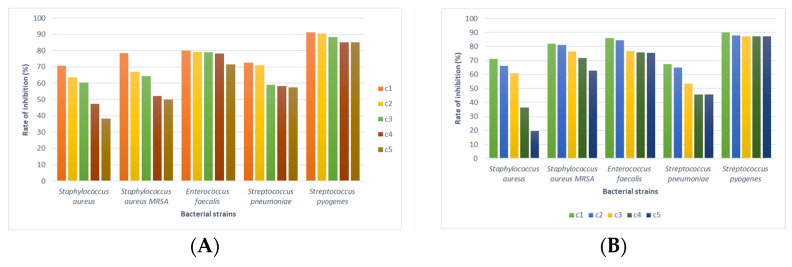
Linden honey antimicrobial activity on Gram-positive ATCC strains (c1—100%, c2—80%, c3—60%, c4—40%, c5—20%), (**A**).—honey sample A (near the village of Tincova), (**B**).—honey sample B—(near the town of Nadrag).

**Figure 5:**
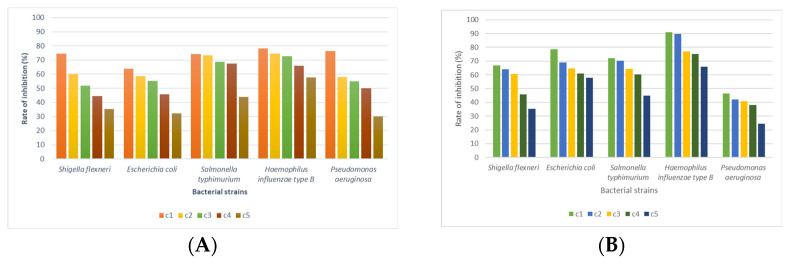
Linden honey antimicrobial activity on Gram-negative ATCC strains (c1—100%, c2—80%, c3—60%, c4—40%, c5—20%), (**A**).—honey sample A (near the village of Tincova), (**B**).—honey sample B—(near the town of Nadrag).

**Figure 6:**
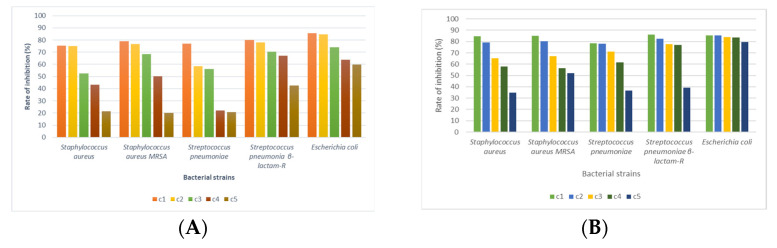
Linden honey antimicrobial activity on bacterial strains isolated from the patient (c1—100%, c2—80%, c3—60%, c4—40%, c5—20%), (**A**).—honey sample A (near the village of Tincova), (**B**).—honey sample B—(near the town of Nadrag).

**Figure 7:**
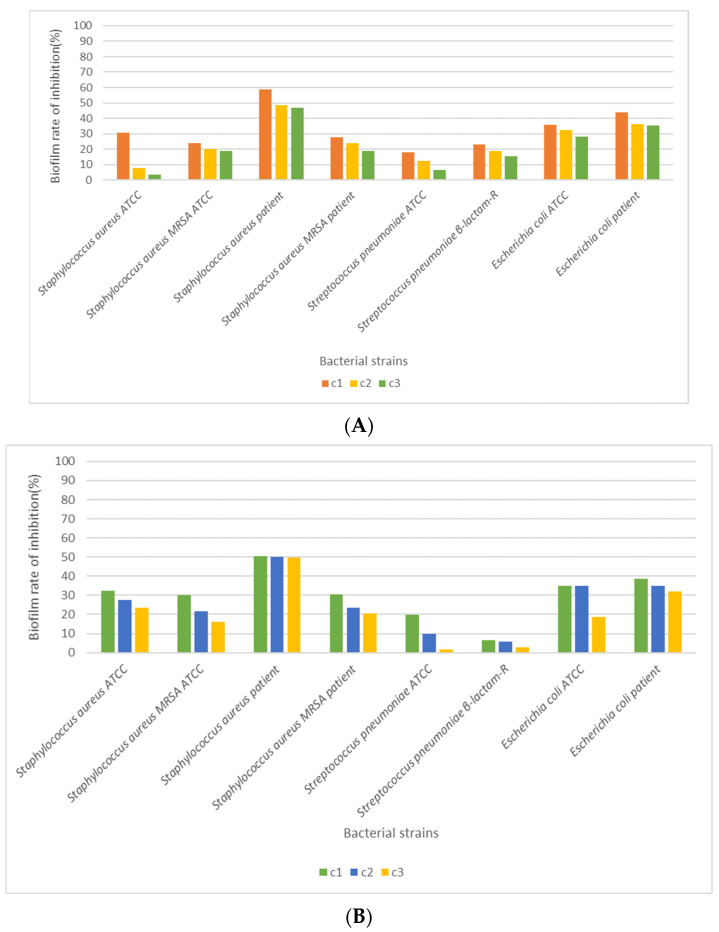
Antibiofilm capacity of linden honey on bacterial strains (c1—100%, c2—80%, c3—60%) (**A**).—honey sample A (near the village of Tincova), (**B**).—honey sample B—(near the town of Nadrag).

The linden honey samples at the established concentrations were applied to the bacterial strains of interest; the values of the bacterial cell inhibition rates were calculated based on the optical density (OD) values, centralized, and displayed graphically (Figure 4, Figure 5 and Figure 6). Also, the ability of linden honey samples to inhibit biofilm formation was determined both in standardized bacterial strains and in strains isolated from patients (Figure 7). The results of the antimicrobial tests were obtained through a spectrophotometric method and consisted of determining the inhibition rate exerted by linden honey on Gram-positive, Gram-negative, and those isolated from patients.

Analyzing the effect of linden honey sample A on standardized Gram-positive bacterial strains, we found that inhibition rates between 91.28% for *S. pyogenes* (c1) and 38.36% for *S. aureus* (c5) are recorded. In the case of linden honey sample B, the inhibition rates on standardized Gram-positive bacterial strains ranged between 90.16% for *S. pyrogenes* (c1) and 19.57% for *S. aureus* (c5). The antimicrobial effect of linden honey decreases for all 5 bacterial strains with the decrease in the tested concentration due to the decrease in the content of polyphenols and flavonoids, compounds responsible for antimicrobial activity.

In the case of the bacterial strains *S. pyogenes* (ATCC 19615), *E. faecalis* (ATCC 29212), and *S. pneumoniae* (ATCC 49619), the values of the inhibition rates highlight a bacteriolytic effect at all 5 tested concentrations for sample A of the linden honey. The values of the inhibition rates recorded for the above-mentioned strains were 85.13–91.28% for *S. pyogenes*, 71.63–79.98% for *E. faecalis*, and 57.50–72.58% for *S. pneumoniae*. In the case of linden honey sample B, the values of the inhibition rates highlight a bacteriolytic effect at all 5 tested concentrations for *S. aureus* MRSA (ATCC 43300), *E. faecalis* (ATCC 29212), and *S. pyogenes* (ATCC 19615) (Figure 4A). The values of the inhibition rates recorded for the above-mentioned strains were 87.37–90.16% for *S. pyogenes*, 75.34–85.93% for *E. faecalis*, and 62.83–81.96% for *S. aureus* MRSA. In the case of applying the cell viability test, based on inhibition rate values above 50%, a bacteriolytic effect of the tested substances is considered, while inhibition rate values below 50% highlight a bacteriostatic effect. The antimicrobial potential of linden honey from sample A in Gram-positive strains decreases as follows: *S. pyogenes* (ATCC 19615) > *E. faecalis* (ATCC 29212) > *S. pneumoniae* (ATCC 49619) > *S. aureus* MRSA (ATCC 43300) > *S. aureus* (ATCC 25923); for sample B, the order was as follows: *S. pyogenes* (ATCC 19615) > *E. faecalis* (ATCC 29212) > *S. aureus* MRSA (ATCC 43300) > *S. aureus* (ATCC 25923) > *S. pneumoniae* (ATCC 49619) (Figure 4B).

The linden honey from sample A determined Gram-negative bacteria inhibition rates between 78.30% for *H. influenzae* type B (c1) and 30.11% for *P. aeruginosa* (c5) (Figure 5A). Linden honey from sample B determined inhibition rates between 90.92% for *H. influenzae* type B (c1) and 24.53% for *P. aeruginosa* (Figure 5B). The bacteriolytic effect was identified for *H. influenzae* type B at all 5 tested concentrations, with the values of the inhibition rates ranging between 78.30 and 57.79% for linden honey sample A and 90.92–66.05% for linden honey sample B. The antimicrobial effect of linden honey tested on Gram-negative bacterial strains decreases as the concentration decreases.

The bacteriolytic effect is maintained at the first 4 concentrations of linden honey from sample A for *S. typhimurium* and *P. aeruginosa* and at the first 3 concentrations tested for *E. coli* and *S. flexneri*. In the case of linden honey from sample B, the bacteriolytic effect was evident at the first 3 concentrations for *S. flexneri*, *E. coli* and *S. typhimurium*. In the case of the bacterial strain *P. aeruginosa*, the effect of linden honey sample B was bacteriostatic, with inhibition rate values below 50%. The antimicrobial potential of linden honey sample A for Gram-negative strains decreases as follows: *H. influenzae* type B (ATCC 10211) > *S. typhimurium* (ATCC 14028) > *P. aeruginosa* (ATCC 27853) > *E. coli* (ATCC 25922) > *S. flexneri* (ATCC 12022) (Figure 5A). Linden honey sample B determined an antimicrobial effect for Gram-negative strains that decreases as follows: *H. influenzae* type B (ATCC 10211) > *E. coli* (ATCC 25922) > *S. typhimurium* (ATCC 14028) > *S. flexneri* (ATCC 12022) > *P. aeruginosa* (ATCC 27853) (Figure 5B).

Comparatively analyzing the antimicrobial effect of linden honey for Gram-positive and Gram- negative bacterial strains, we found, based on the values of the inhibition rates, a more obvious effect for Gram-positive bacteria, with the differences being statistically significant (*p* < 0.05).

The values of the inhibition rates calculated for the bacterial strains isolated from patients were 85.69% *E. coli* (c1) and 20.84% *S. pneumoniae β-lactam-R* (c5) in linden honey sample A (Figure 7A), and 86.22% *S. pneumoniae β-lactam-R* (c1) and 34.70% *S. aureus* (c5) in linden honey sample B (Figure 7B). The decrease in the antibacterial potential of linden honey is evident depending on the bacterial strain and the tested concentration.

Comparing based on the values of the inhibition rates, the effect of linden honey on the bacterial strains isolated from patients with the one recorded for Gram-positive bacteria, the effect is lower (*p* < 0.05), and compared to the Gram-negative ones, it is similar.

The testing of linden honey’s ability to inhibit biofilm formation was performed only for the first 3 concentrations of honey; their choice is due to the fact that the antimicrobial effect determined by them was bacteriolytic for all the strains studied. Both standardized bacterial strains were selected as follows: Gram-positive—*S. aureus* (ATCC 25923), *S. aureus* MRSA (ATCC 43300), and *S. pneumoniae* (ATCC 49619); Gram-negative—*E. coli* (ATCC 25922); and strains isolated from patients (*S. aureus*, *S. aureus* MRSA, *S. pneumoniae β-lactam-R*, *E. coli*).

The ability of linden honey to inhibit biofilm formation in the selected strains was most evident in *S. aureus* isolated from the patient, with the recorded values being between 58.57 and 46.84% for sample A (Figure 7A) and 50.56–49.55% for sample B. The lowest values were recorded for *S. pneumoniae* ATCC, ranging between 19.76 and 1.68% for honey sample A and 6.64–2.89% for honey sample B (Figure 7B). In the case of the strain of *E. coli*, the ability to inhibit biofilm formation is more evident in the strain isolated from the patient compared to the standardized one. However, a similar situation was found in the case of the strain of *S. pneumoniae β-lactam-R* regarding the ability to inhibit biofilm formation by linden honey. In the case of the *S. aureus* strain, the ability of linden honey to inhibit biofilm formation is more evident for the strain isolated from the patient compared to the MRSA strain.

## 4. Discussion

Honey has a complex composition; approximately 95% of its dry matter is composed of carbohydrates [55], mainly glucose and fructose [56]. In addition to sugars, honey contains up to 20% water [57] and a number of other active compounds such as proteins (enzymes), organic acids, vitamins (especially vitamin B6, thiamin, niacin, riboflavin, and pantothenic acid), minerals (potassium, calcium, magnesium, copper, iron, manganese, phosphorus, sodium, and zinc), pigments, phenolic compounds, and a wide variety of volatile compounds and derived solid particles, which contribute to the quality and health benefits of honey [4,5]. The chemical composition of honey and its quality are influenced by several factors, including floristic composition, geographical area, weather conditions, humidity inside the hive, nectar quality, treatment applied to the bees and/or handling methods used during the extraction, and the storage process.

The water content of honey is a quality criterion that influences the fluidity of honey, the crystallization process, and some textural properties such as adhesiveness [58]. A reduced water content inhibits the development of microorganisms, which prevents the fermentation of honey and extends their shelf life, an aspect that provides information about the degree of maturation and its quality [59,60]. In our study, the water content of the tested honey samples was determined using the conventional oven-drying method, with the average value being 15.23% (sample A) and 19.19% (sample B) (Table 2). The result obtained is in accordance with the EU directive 2001/110, according to which the moisture content of honey should not exceed the threshold of 20% [61].

Impurities can enter honey at various stages, including during the processing and packaging process. The impurity content of the samples tested by us has an average value of 71 mg/100 g honey and an ash content value of 0.48%. According to European legislation, the impurity content of honey must be below 100 mg/100 g of honey, and the total ash content must not exceed 0.6% for flower nectar honey and below 1.2% for honeydew honey. In our study, the impurity content was 71 mg/100 g honey (sample A) and 68 mg/100 g honey (sample B), values that are within range according to European legislation. Our values regarding the impurity content are higher compared to those presented in another study conducted on honey varieties from Romania [24]. The acid content of honey is relatively low, but it is important for imparting taste and maintaining pH. The acidity of the linden honey samples, tested using the titration method, has an average value of 4.84 mg/100 g (sample A) and 6.10 mg/100 g (sample B), and the pH value was 3.51 (sample A) and 3.53 (sample B). According to EU directive 2001/110 EC, the acidity of honey must be below 50 meq/kg. The presence of different organic acids such as formic, acetic, citric, lactic, maleic, malic, oxalic, pyroglutamic, and succinic acids, the geographical origin, and the harvest season can influence its value [62,63]. Most flower honeys have an acidic pH, which varies between 3.3 and 4.6, with the exception of chestnut honey, which has a pH value of 5 to 6. In addition, honeydew honey, due to its higher mineral content, has a pH value between 4.5 and 6.5. Due to its content of phosphates, carbonates, and other mineral salts, honey has buffer properties, which means that its pH remains unchanged if small amounts of acids and bases are added [63]. A unique characteristic of honey is the formation of granules, which sets it apart from other sweeteners. The pH value influences the texture and stability of honey [64]. The acidity values recorded in the linden honey samples in our study are comparable to those determined in other studies, perhaps slightly increased for the tested honey sample B [24,64].

Previous studies have shown a close relationship between the phenolic profile of different honey varieties, antioxidant capacity, geographical origin, and floral source [65,66,67]. Regarding the content in phenolic compounds (PC), the honey analyzed in this study had an average value of 862.32 mg/kg (sample A) and 871.33 mg/kg (sample B). Comparing the phenolic compounds values recorded in our study with those recorded in other studies that targeted linden honey from apiaries in Romania, we found that the values are slightly higher in our samples compared to linden honey samples from Timisoara [24], and four times higher than in honey samples from Sanmihaiu Roman [25].

In our samples, DPPH value was 80,39% (sample A) and 77.51% (sample B). For honey from Spain, the DPPH value was 52.9–95.6% [68]; for honey from Greece, the value was 56.8–72.4% [69] and 12.2–48.89% for honey from Croatia [70]. The previously mentioned studies report a higher value of DPPH for dark-colored honey (linden honey, honeydew honey) compared to light-colored honey (acacia honey, rapeseed honey), where the recorded values are lower. Comparing the DPPH values of the linden honey samples analyzed by us with those obtained in the studies from Greece and Croatia, the values recorded in the linden honey samples from Romania were higher, indicating a higher antioxidant activity. Similarly, increased DPPH values were recorded when comparing our values with those obtained for linden honey from apiaries in Romania, located in different geographical regions [24,25]. The mineral content of honey is an important parameter in assessing the nutritional value of the product and in the authentication and classification of honey according to the botanical and geographical origin of the nectar [1,71,72,73,74]. In addition, the mineral composition of honey can also vary depending on climatic conditions, soil characteristics, bee species, honey maturation process, and colony health. From the chemical analysis of the mineral content of the linden honey samples taken by us from the hilly and mountain area of Banat, we observed a variability of the macro- and micromineral elements. Potassium is the dominant mineral element with a weight of 93.52 mg/kg (sample A) and 111.77 mg/kg (sample B), followed by calcium 78.37 mg/kg (sample A) and 92.93 mg/kg (sample B), and magnesium 39.12 mg/kg (sample A) and 49.23 mg/kg (sample B). Storage conditions, installation of the crystallization process, content in minerals, and flavonoids and phenolic compounds influence the color of honey; dark honey has a higher mineral content than light honey [75,76]. The highest mineral content was detected in linden honey, polyflora honey, and honeydew honey, with the predominant being potassium; copper values were between 46.35 and 466.69 mg/kg, magnesium 5.71–72.31 mg/kg, and sodium 6.75–160.04 mg/kg [77]. Comparing the concentrations of K, Ca, and Mg in linden honey from our samples with the values presented in other studies, comparable values were found [24,72,73]. Fe, Zn, and Mn are trace elements with important physiological implications; in our samples, their average value is for iron 9.37 mg/kg (sample A) and 15.51 mg/kg (sample B), zinc 4.15 mg/kg (sample A) and 15.10 mg/kg (sample B), and manganese 1.294 mg/kg (sample A) and 0.425 mg/kg (sample B). The specialized literature reported iron values between 0.79 and 5.51 mg/kg, for copper 0.53 1.6 mg/kg, manganese 0.21–7.97 mg/kg, and zinc 0.38–20.36 mg/kg [77]. We can observe that the values presented in the literature are comparable to those determined in our samples. Soil, water, air, and plants are visited by bees and are brought to the hive in the form of pollen, nectar, propolis, water and, with them, pollutants. These pollutants accumulate in the body of the bee (due to its vital activity) and in its products (honey, pollen, propolis). Pollutants such as heavy metals, including Pb, Cd, Hg, Cr, Cu, Ni, have an extremely harmful impact on the environment, being emitted into the atmosphere in the form of fine powders, and at high temperatures, in the form of gases, from where they are gradually taken up by both plants and insects and animals. The presence of high concentrations of heavy metals in ecological systems can cause serious health implications and depends on its botanical origin, the type of soil, and the anthropogenic activities that take place in that area [78,79]. According to the Agency for Toxic Substances, the most dangerous heavy metals are Pb, As, and Cd. Studies have shown that As is less present in honey, but Pb contamination has been frequently reported [79].

Following the analyses carried out by our team, we found that the Pb content in the linden honey samples has an average value of 0.037 mg/kg (sample A) and 0.076 (sample B), and is influenced by the specifics of the area where the apiary was located, namely, the hilly area of Banat. Similar results were also obtained by Zugravu et al. [80] following the analysis of honey samples from different regions of Romania, where the average concentration of Pb in honey was 0.1209 ± 0.0445 mg/kg in unpolluted areas and 0.1907 ± 0.0437 mg/kg in polluted areas. These results underline the fact that the average concentrations of Pb in the honey tested in this study do not exceed the LMA established by the EU and FAO standards of 0.20 mg/kg. Numerous other studies reported a lead content value of 0.06–0.13 mg/kg for honey from Poland [81], 0.09–0.36 mg/kg for honey from Croatia [82], and 0.378 mg/kg for honey from France [83]. In Argentina, Brazil, and Venezuela, research has shown that honey has a Pb content of 0.11–0.33 mg/kg [84], for Turkish honey of 0.05–0.48 mg/kg, and for Egyptian honey 0.09–0.64 mg/kg [85].

Looking at the Cd residues in our honey samples, they had an average value of 0.016 mg/kg (sample A) and 0.057 mg/kg (sample B). Other researchers reported an average value of Cd in honey samples of 0.0103 ± 0.0027 mg/kg for unpolluted areas versus 0.0167 ± 0.0012 mg/kg for industrial areas and those located near the highway [80]. The level of Cd reported in Iran was 0.013 mg/kg [86] in the industrial area [87], and in Macedonia, the reported level of Cd was 0.027 mg/kg [86]; in Italy, it was 0.020 mg/kg [88]. The chromium content of our samples had an average value of 0.110 mg/kg, and the average value of the nickel content was 0.257 mg/kg.

Honey has a high content of sugars, responsible for its nutritional value, ability to crystallize, and viscosity; the main ones are monosaccharides of the glucose and fructose type, which vary between 60 and 85% of the dry matter of honey [1,89]. In mint, rapeseed, sunflower, honeydew honey, and some pasture honey samples, the sugar that was found in the highest amount was glucose; and for polyflora honey, acacia, lime, raspberry, dandelion, chestnut, and some pasture honey samples, the higher concentration was fructose [90]. For our samples, the amount of reducing sugar was 66.83% (sample A) and 71.74% (sample B). According to EU directive 2001/110 EC, for floral honey the lowest amount for reducing sugar is 60 g/100 g ^−1^ and 45 g/100 g ^−1^ for honeydew honey. The recorded values are consistent with European legislation and with those recorded in other studies conducted on linden honey [24].

The flavonoid content of the honey samples analyzed in this study recorded values ranging between 242.34 mg QE/100 g (sample A) and 268.89 mg QE/100 g (sample B). The recorded values are comparable to those determined in other studies on the flavonoid content of linden honey [24,25]. In honey, flavonoids come from nectar, pollen, and propolis. The flavonoid content depends on the botanical origin and the year of harvest [14,66]. Flavonoids, together with other compounds from the phenol group, contribute to protection against free radicals, which justifies their anti-inflammatory and antioxidant effects [66,68]. Among all natural products, honey, due to its properties, is widely used for various applications, including food, cosmetics, medicine, and in various cultural practices around the world. However, special attention is paid to its therapeutic properties, as honey exhibits antimicrobial potential. This aspect is of particular importance considering the increasingly frequent resistance of bacterial strains to antibiotics and the expansion of the number of bacterial species involved in nosocomial infections. Currently, the emerging trends of antimicrobial resistance of bacterial pathogens at the wound level are a serious challenge. Thus, honey, with effective antimicrobial properties against antibiotic-resistant organisms such as MRSA and MDR, is in great demand [91].

The obvious antibacterial and antibiofilm effects are the major attributes of the so-called “medical grade honey”, which is used topically for the treatment of burns, wounds, and skin conditions [92]. The antibacterial and antibiofilm activities of different types of honey on different groups of bacteria is well documented in the literature. However, we chose to continue this line of research because many of the results are contradictory.

In this sense, a study carried out shows a positive effect of stimulating microorganisms, but with negative values for *S. aureus*, *P. aeruginosa*, *S. typhimurium*, *H. influenzae*, and *C. parapsilopsis*. The value of the inhibition rate increased with increasing concentration, but they did not reach the minimum inhibitory concentration (MIC). These results were recorded at a linden honey concentration of 25% for *S. pyogenes*, *E. coli*, *L. monocytogenes*, and *B. cereus* [24]. The antimicrobial effects of linden honey, which are also contradictory, indicate low antimicrobial activity against *S. pyogenes*, *S. aureus*, *P. aeruginosa*, and *S. typhimurium*, but good antimicrobial activity against *S. flexneri*, *E. coli*, and *H. influenzae* [25]. In other studies, the authors reported average MIC values for linden honey of 7.3% for *S. aureus* and 11.5% for *P. aeruginosa* [93]. In contrast, high antimicrobial activity was observed against the *S. pneumoniae* strain (21.3–42.5%) [36].

The compound with a major antibacterial role in many varieties of honey is H_2_O_2_, but different factors dependent on bees or plants seem to contribute to the antimicrobial and antibiofilm effect of honey. Among these substances, we mention defensin-1, osmolarity, and some protein molecules. It is generally known that dark-colored honey often produces higher amounts of H_2_O_2_ than light-colored honey [94], but in the study by Farkasovska and her research team, a very weak correlation was found between the hydrogen peroxide content of honey and its antibacterial activity.

Linden honey is a light-colored honey. The antibiofilm activity is similar to the dark-colored chestnut honey, but remarkable amounts of minerals are evident in both types of honey [93]. According to studies carried out by groups of researchers, it was concluded that darker colored honey has a higher antibacterial potential, a fact that is partly related to the concentration of H_2_O_2_. However, some research teams did not observe the correlation between H_2_O_2_ concentration and antibacterial activity of Greek pine honey samples [95] or some honey varieties from Slovakia [35].

The content of flavonoids in honey samples can influence its antimicrobial and antibiofilm potential. Thus, the presence of higher amounts of flavonoids in Brazilian honey or Borneo bee honey could contribute to their stronger antimicrobial activity. In this study, it was hypothesized that the high antibacterial and antibiofilm activities of linden and chestnut honey are at least partially due to the higher level of non-peroxidic compounds, mainly flavonoids [34].

In several studies, differences were observed regarding the antimicrobial activity of honey in Gram-positive bacteria compared to Gram-negative ones. The results of our studies are similar to the findings of other research groups. Conversely, honey samples from the Brazilian semi-arid region, with the highest phenolic content, showed the lowest MIC values against the tested bacterial strains, which supports the main role of non-peroxidic compounds (polyphenolics and flavonoids) with antibacterial effect [34].

The linden honey analyzed in this study showed different antimicrobial effects depending on the tested concentration and the type of bacterial strain on which it was applied. The anti-microbial effects were more evident in Gram-positive bacteria compared to Gram-negative ones, for sample A by 18.37%, and for sample B by 15.72%. Also, in standardized Gram-positive bacteria the effect was more evident compared to those isolated from patients, for sample A by 15.72%, and for sample B by 1.32%. The antibiofilm potential of linden honey was highlighted in all bacterial strains studied, with the effect being more evident in bacteria isolated from patients compared to standardized strains: for honey sample A by 66.95% and for honey sample B by 54.67%. It is worth noting a less evident antibiofilm effect in bacteria with antibiotic resistance compared to the other bacterial strains: 7.01% lower values for honey sample A and 8.80% lower values for sample B. In a study on the effect of 11 varieties of honey from Romania, the antimicrobial effect of linden honey was also analyzed. The results indicated a negative effect of increasing tension; therefore, the effect decreases with increasing concentration. In the case of *S. flexneri*, linden honey with a concentration of 10% showed a value of BIR of −60.37%, and at a concentration of 25%, the value obtained was −179.66%, so the honey presented a stimulation of the development of the strain of microorganisms. Although the results obtained were positive, the trend shown was negative: the lower concentration determines a better effect, which implies the synergistic effect of the components of linden honey on the strains of microorganisms [24]. In our study, antimicrobial activity of linden honey tested against the *S. flexneri* strain demonstrated notable antibacterial potential. At the first three tested concentrations, inhibition rates ranged from 51.97% to 74.63% for sample A and from 60.63% to 66.92% for sample B. These results confirm a pronounced antibacterial effect at higher concentrations, particularly those approaching the honey’s natural form. Interestingly, our findings contradict those of the previously reported study, which suggested that elevated honey concentrations may stimulate bacterial growth. Another study captures the effects of some varieties of Hungarian honey on the essential bacteria of the respiratory tract. The obtained results are the first to demonstrate the bioactivity of honey against *H. parainfluenzae*. The inhibitory effect of linden honey on *S. pneumoniae* was also highlighted. The study highlights that honey of different botanical origins can offer an alternative means of treating respiratory tract infections due to their potential to reduce bacterial growth and biofilm formation. Among the varieties of investigated Hungarian honey, linden honey, especially, deserves more attention, proving to be a strong natural antibacterial agent [36]. The linden honey samples tested in this research exhibited inhibitory effects against *S. pneumoniae* across all five concentrations, with average inhibition rates of 63.69% for sample A and 55.47% for sample B. Similarly, antibacterial activity against *H. influenzae* was observed at all tested concentrations, with sample A showing an average inhibition rate of 69.90% and sample B reaching 79.81%. Our study’s results align with those reported in the previously cited research and further validate the antimicrobial properties of linden honey against bacterial strains commonly associated with respiratory tract infections.

A separate study investigating the antibacterial potential of linden honey sourced from an apiary in western Romania reported low antimicrobial activity against *S. pyogenes*, *S. aureus*, *P. aeruginosa*, and *S. typhimurium*, while showing moderate activity against *S. flexneri*, *E. coli*, and *H. influenzae*—though all inhibition values remained below 40% of the MIC [25]. In contrast, the linden honey samples analyzed in our study, also originating from an apiary in western Romania, exhibited higher average inhibition rates across five tested concentrations: 56.06% (sample A) and 50.91% (sample B) for *S. aureus*; 65.59% (sample A) and 62.41% (sample B) for *S. typhimurium*; and 51.15% (sample A) and 66.25% (sample B) for *E. coli*. These discrepancies likely stem from differences in the botanical and geographical origin of honey, which influence its physicochemical properties and, consequently, its antibacterial efficacy. Honey, due to its components, which act synergistically, can be used in a variety of applications, with the medical ones being of major interest. The antimicrobial and antibiofilm activities of honey are closely related to its chemical composition, which in turn is influenced by endogenous factors (botanical origin), but especially exogenous factors (geographic area where beehives are located, climatic conditions, technological process of harvesting, processing, transport, and storage). As a result, each variety of honey represents a unique natural source that must be characterized from the point of view of its physico-chemical, anti-inflammatory, antioxidant, antimicrobial, and antitumor properties [96,97].

The physico-chemical properties of honey are decisive factors in establishing the quality, in its choice by the consumer, as well as in the management of good marketing. For linden honey, from the hilly area, all the chemical parameters tested in this study were in accordance with the International Standards for honey stipulated in EU Directive 2001/110 EC. The optimal values of these parameters suggest a higher quality honey, handled and stored in appropriate conditions. Periodic testing and monitoring of honey quality properties based on geographical origin and in accordance with standard procedures are essential to maintain the natural quality of the product for a long time and at the same time to control the widespread practice of its adulteration.

The results presented in this study come from the analysis of a single honey variety—linden honey, originating from two different locations and included a characterization of the honey only in terms of physicochemical characteristics, antimicrobial, and antibiofilm potential. In the future, we intend to expand our studies by analyzing new honey varieties, with different botanical and geographical origins. We intend to continue the directions of analysis presented in the current study, and future studies will also be completed with the assessment of other properties of honey through analyses that highlight the anti-inflammatory, antioxidant, antitumor potential, etc.

## 5. Conclusions

The physical characteristics, chemical composition, and antimicrobial properties, including antibacterial and antibiofilm activity, of the analyzed linden honey samples varied according to the botanical origin and geographical location of the apiary. Overall, sample B demonstrated stronger antibacterial and antibiofilm effects compared to sample A, with inhibition rates exceeding those of sample A by 6.54% and 8.71%, respectively. These enhanced effects are attributed to sample B’s higher concentrations of phenolic compounds (871.33 mg/kg), flavonoids (268.22 mg QE/100 g), as well as elevated pH (3.53) and acidity levels (6.10). Based on the observed inhibition of bacterial growth and biofilm formation, the tested linden honey exhibits promising antimicrobial potential and may serve as a natural therapeutic agent for a broad spectrum of conditions.

## Figures and Tables

**Table 2:** Physico-chemical properties and mineral content of linden honey from the hilly area of Banat: water content (WC) (%), impurities (IC) (%), ash (AC) (g/100 g), acidity (A) (VNaOH) (mL), pH, reducing sugars (RS) (%), DPPH (%), and phenolic compounds (TPC) (mg/kg).

		Sample A			Sample B			
Parameter		Mean ± Standard Deviation (x ± SD)	Min	Max	Mean ± Standard Deviation (x ± SD)	Min	Max	*p*
Water content (WC)(%)		15.23 ± 0.03	15.21	15.26	19.21 ± 0.02	19.19	19.22	<0.0001
Impurities (IC) (mg/100 g)		71 ± 0.01	71	72	68 ± 0.01	67	68	0.0015
Ash (AC) (g/100 g)		0.48 ± 0.06	0.42	0.53	0.47 ± 0.03	0.45	0.50	0.7504
Acidity (A) (VNaOH) (mL)		4.84 ± 0.11	4.87	4.93	6.10 ± 0.05	6.05	6.15	<0.0001
pH		3.51 ± 0.07	3.45	3.58	3.53 ± 0.07	3.48	3.48	0.6634
Reducing sugars (RS) (%)		66.83 ± 0.04	66.79	66.87	71.74 ± 3.21	69.38	73.41	0.0268
DPPH (%)		80.39 ± 0.02	80.37	80.41	77.51 ± 3.21	75.17	81.17	0.1153
Phenolic compounds (TPC) (mg/kg)		862.32 ± 0.88	861.56	863.28	871.33 ± 0.00	871.33	871.33	<0.0001
Flavonoid content (FC) (mg QE/100 g)		242.34 ± 0.59	241.75	242.93	268.22 ± 0.94	268.89	267.56	<0.0001

**Table 3:** Results of PC_1_ and PC_2_ principal components and corresponding vectors for honey physicochemical properties: water content (WC) (%), impurities (IC) (%), ash (AC) (g/100 g), acidity (A) (VNaOH) (mL), pH, reducing sugars (RS) (%), DPPH (%), and phenolic compounds (TPC) (mg/kg).

Principal Components	Sample A		Sample B	
	PC_1_	PC_2_	PC_1_	PC_2_
Eigen value	6.192	1.808	4.445	2.552
% of variance	77.399	22.601	63.540	36.459
Parameters	Vector1	Vector2	Vector1	Vector2
WC	−0.398	0.098	−0.429	−0.267
IC	0.402	−0.013	−0.471	−0.067
AC	−0.398	−0.103	−0.203	0.566
A	0.388	−0.191	0.051	−0.622
pH	−0.391	0.174	0.459	0.156
RS	−0.254	−0.576	0.361	−0.407
DPPH	0.285	−0.525	0.457	0.164
TPC	0.269	0.556	0	0

**Table 4:** Results of the principal components PC_1_ and PC_2_ and the corresponding vectors for the mineral composition of the analyzed linden honey. (Copper (Cu), Nickel (Ni), Chromium (Cr), Lead (Pb), Zinc (Zn), Iron (Fe), Manganese (Mn), Calcium (Ca), Magnesium (Mg), Potassium (K), and Copper (Cu)).

Principal Components	Sample A		Sample B	
	PC_1_	PC_2_	PC_1_	PC_2_
Eigen value	8.935	2.066	6.925	4.075
% of variance	81.223	18.777	62.957	37.043
Parameters	Eigen-Vector1	Eigen-Vector2	Vector1	Vector2
Cd	0.327	−0.146	0.230	0.394
Cu	−0.335	0.015	0.354	−0.179
Ni	−0.327	0.146	0.296	0.310
Cr	−0.335	0.015	−0.058	0.489
Pb	0.007	0.696	−0.319	−0.269
Zn	0.319	−0.207	−0.354	0.181
Fe	0.286	−0.361	0.178	−0.438
Mn	0.323	0.179	0.366	−0.132
Ca	0.323	0.179	−0.336	−0.232
Mg	0.246	0.472	−0.376	−0.076
K	−0.329	−0.117	−0.289	0.321

Note: only the principal components and their vectors are included in the table.

## Data Availability

The report of the analyses performed for the samples in the paper can be found at the Interdisciplinary Research Platform (PCI) belonging to the University of Life Sciences “King Mihai I” from Timisoara, Romania. The original contributions presented in this study are included in the article/Supplementary Materials. Further inquiries can be directed to the corresponding authors.

## Supplementary Materials

The following supporting information can be downloaded at https://www.mdpi.com/article/10.3390/foods14213594/s1. Palynological analysis of the tested honey samples can be found in Supplementary Material S1—Figure S1. Different types of pollen identified in the honey samples.

## Abbreviations

The following abbreviations are used in this manuscript:

WC	Water content
IC	Impurities
AC	Ash
A	Acidity
RS	Reducing sugars
TPC	Phenolic compounds
PCA	Principal component analysis
Cu	Copper
Ni	Nickel
Cr	Chromium
Pb	Lead
Fe	Iron
Zn	Zinc
Mn	Manganese
Ca	Calcium
Mg	Magnesium
K	Potassium
