# Supplementary material for: Antimicrobial and Biofilm Inhibiting Potential of Two Romanian Linden Honeys
Source: Foods. 2025 Oct 22;14(21):3594. doi: 10.3390/foods14213594 (PMC12607591; doi:10.3390/foods14213594)
Supplement: Supplementary file 1 [file foods-14-03594-s001.zip › foods-3907463-supplementary.pdf]

Antimicrobial and Antibiofilm Potential of Linden Honey as a Natural Product

Alexandru Nan[1,2], Mihai Mituletu[3,4*], Gabi Dumitrescu[2,3*], Ion Valeriu Caraba[2,3], Ioan Pet[2], Adrian Sinitean[5], Mariana Adina Matica[5,6], Petculescu Chiochina Liliana[2], Elena Pet[7], Popescu Roxana[3,4], Marioara Nicoleta Caraba[3,4]

[1] 1 Doctoral School "Engineering of Vegetable and Animal Resources", University of Life Sciences "King Mihai I" from Timişoara, Calea Aradului 119, 300645-Timişoara, Romania, alexandru.nan@usvt.ro (A.N.)
[2] Faculty of Bioengineering of Animal Resources, University of Life Sciences "King Mihai I" from Timisoara, Calea Aradului, 119, Timisoara, 300645, Romania, alexandru.nan@usvt.ro (A.N.); gabidumitrescu@usvt.ro (G.D.); valeriucaraba@usvt.ro (I.V.C.); ioanpet@usvt.ro (I.P.); lilianapetculescuciochina@usvt.ro (L.P.C.)
[3] ANAPATMOL Research Center, "Victor Babes" University of Medicine and Pharmacy of Timisoara, E. Murgu, 2, Timisoara, 300041, Romania, gabidumitrescu@usvt.ro (G.D.); valeriucaraba@usvt.ro (I.V.C.); mihai.mituletu@umft.ro (M.M.); popescu.roxana@umft.ro (R.P); nicoleta.caraba@umft.ro (M.N.C.)
[4] Faculty of Medicine, "Victor Babes" University of Medicine and Pharmacy Timisoara, E. Murgu, 2, 300041 Timisoara, Romania, mihai.mituletu@umft.ro (M.M.); popescu.roxana@umft.ro (R.P.); nicoleta.caraba@umft.ro (M.N.C.)
[5] Department Biology, Faculty of Chemistry-Biology-Geography, West University of Timisoara, Pestalozzi, 16, 300315 Timisoara, Romania; adrian.sinitean@e-uvt.ro (A.S.); mariana.matica@e-uvt.ro (M.A.M.)
[6] Advanced Environmental Research Laboratories (AERL), 4 Oituz, 300086 Timisoara, Romania, adrian.sinitean@e-uvt.ro (A.S.); mariana.matica@e-uvt.ro (M.A.M.)
[7] Faculty of Management and Rural Tourism, University of Life Sciences "King Mihai I" from Timisoara, Calea Aradului, 119, 300645 Timisoara, Romania; elenapet@usvt.ro (E.P.)
* Correspondence: mihai.mituletu@umft.ro (M.M.), gabidumitrescu@usvt.ro (G.D.)

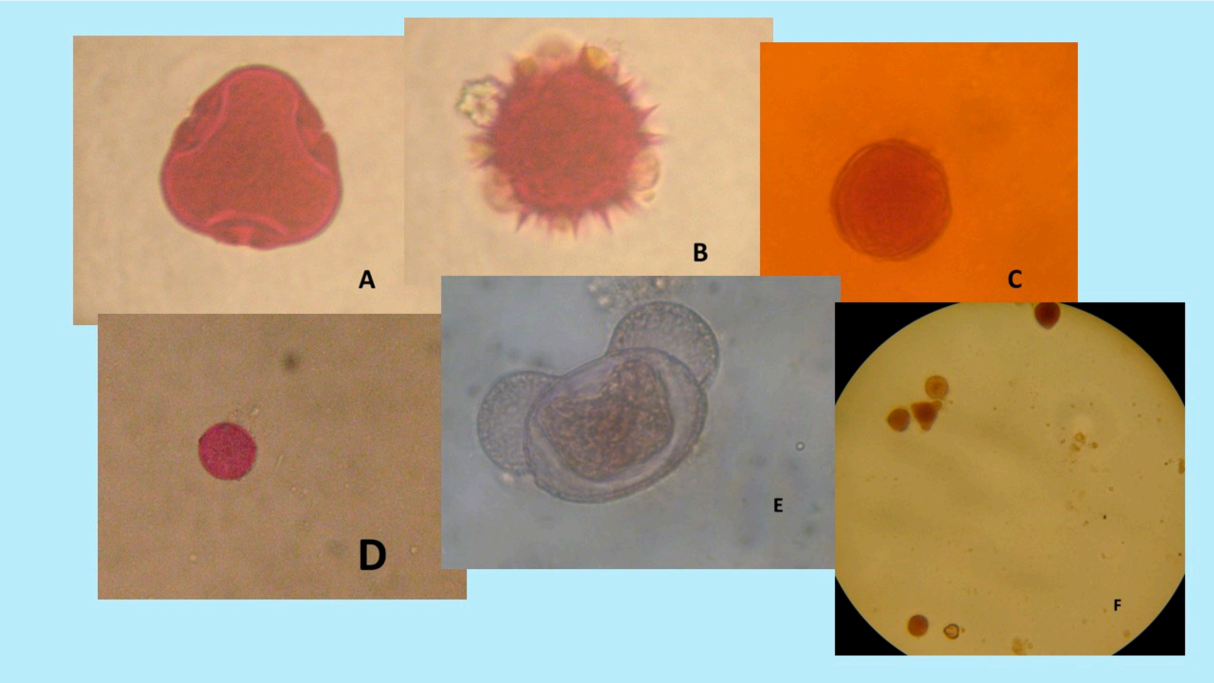

Figure S1. Different types of pollen identified in the honey samples: A – Tilia sp., B – Asteraceae, C – Robinia sp., D – Plantago sp., E – Pinus sp. F – general aspect of a microscopic field with multiple types of pollen; (A, B, C, E – 400x; D, F – 100x)
